# Enteral tolerance in critically ill patients

**DOI:** 10.1186/s40560-019-0378-0

**Published:** 2019-05-07

**Authors:** Hiroomi Tatsumi

**Affiliations:** Department of Intensive Care Medicine, Sapporo Medical University School of Medicine, South 1 West 16, Chuo-ku, Sapporo, Hokkaido 060-8543 Japan

**Keywords:** Enteral nutrition, Tolerance, Intolerance, Gastrointestinal disorders, Diarrhea

## Abstract

Enteral nutrition (EN) can maintain the structure and function of the gastrointestinal mucosa better than parenteral nutrition. In critically ill patients, EN must be discontinued or interrupted, if gastrointestinal complications, particularly vomiting and bowel movement disorders, do not resolve with appropriate management. To avoid such gastrointestinal complications, EN should be started as soon as possible with a small amount of EN first and gradually increased. EN itself may also promote intestinal peristalsis. The measures to decrease the risk of reflux and aspiration include elevation the head of the bed (30° to 45°), switch to continuous administration, administration of prokinetic drugs or narcotic antagonists to promote gastrointestinal motility, and switch to jejunal access (postpyloric route). Moreover, the control of bowel movement is also important for intensive care and management. In particular, prolonged diarrhea can cause deficiency in nutrient absorption, malnutrition, and increase in mortality. In addition, diarrhea may cause a decrease the circulating blood volume, metabolic acidosis, electrolyte abnormalities, and contamination of surgical wounds and pressure ulcers. If diarrhea occurs in critically ill patients on EN management, it is important to determine whether diarrhea is EN-related or not. After ruling out the other causes of diarrhea, the measures to prevent EN-related diarrhea include switch to continuous infusion, switch to gastric feeding, adjustment of agents that improve gastrointestinal peristalsis or laxative, administration of antidiarrheal drugs, changing the type of EN formula, and semisolidification of EN formula. One of the best ways to success for EN management is to continue as long as possible without interruption and discontinuation of EN easily by appropriate measures, even if gastrointestinal complications occur.

## Introduction

The difficulties and complications related with enteral nutrition (EN) include metabolic disorders, such as increase in blood glucose level and electrolyte abnormality, but this article will mainly describe the gastrointestinal complications, particularly vomiting and bowel movement disorders (i.e., diarrhea and constipation), which are frequently observed during EN and are important to address. If such gastrointestinal symptoms do not resolve with appropriate management, EN must be discontinued or interrupted, and parenteral nutrition (PN) must be easily initiated.

### Intestinal intolerance and confirmation of gastrointestinal peristalsis

Regardless of EN administration, several gastrointestinal disorders and symptoms can still occur in critically ill patients and can be precipitated by several factors, including diseases, general condition, and metabolic state before the onset, setting of respirator, and administered drugs [1]. Gastrointestinal disorders may relate to intestinal intolerance during EN. The mechanisms of gastrointestinal disorders in critically ill patients or postoperative patients can be classified as failure of mucosal barriers, attenuation of gastrointestinal peristalsis and atrophy of intestinal mucosa, decrease of gut-associated lymphatic tissue and so on [2].

Previously, ingestion can be started once peristalsis, bowel movement, or flatus is confirmed. Currently, however, early EN within 48 h of intensive care unit (ICU) admission may be initiated safely without the confirmation of these signs [3–7]. In fact, guidelines recommended that the decision to initiate EN should not be based on the confirmation of gastrointestinal peristalsis [8].

The presence of bowel sounds had been commonly used as a criterion for EN initiation. However, bowel sounds reflect gas movement in the intestines, and almost the same sounds can be heard when water and gas are injected via a tube inserted to the duodenum [9]. In other words, the bowel sounds cannot confirm normal functioning of the intestines, intestinal integrity, mucosal barriers, and preserved intestinal absorption. Therefore, it is important to start with a small amount of EN first, because bowel sounds cannot be used as a basis for EN initiation. Conversely, EN should be started as soon as possible, because EN itself may promote intestinal peristalsis.

There are other reasons to support early EN. The early initiation of EN including fiber may prevent atrophy of intestinal mucosa and attenuation of gastrointestinal peristalsis, because the energy substrates for intestinal mucosa are partially supplied via intraluminally. Moreover, it is believed that the early initiation of EN may prevent bacterial translocation (BT).

### Monitoring of intestinal intolerance

The symptoms of patient intolerance to EN vary. It is important to comprehensively monitor for pain, abdominal distension, other clinical symptoms and findings, bowel movement or flatus, and abdominal X-ray, in order to guide the decision to continue EN or not and to avoid inappropriate discontinuation of EN [8].

Gastric residual volume (GRV) had been shown to not correlate well with the incidence of pneumonia [10–12], gastric emptying ability [13–15], and incidence of regurgitation and aspiration [16]. Decreasing the cutoff value of GRV cannot avoid such complications and may lead to inappropriate interruption, discontinuation, or reduction of the amount of EN administered [10]. Even if the GRV is less than 500 mL, EN should not be interrupted without any symptoms that indicate intestinal intolerance [17]. If GRV is restricted within 200–500 mL, EN should be carefully continued and measures to decrease the risk of aspiration should be taken, as described later.

Reignier et al. [18] reported that GRV monitoring (under 250 mL) did not affect the mortality and incidence of ventilator-associated pneumonia and infection. Poulard et al. [19] reported the same results for the incidence of complications, but the incidence of intestinal intolerance was significantly lower in the group without GRV monitoring. Regarding the criteria for GRV measure, four randomized controlled trials (RCTs) showed that the incidences of reflux, aspiration, and pneumonia did not increase when the cutoff for GRV was increased from 50–150 mL to 250–500 mL [10–12, 17]. In addition, although the GRV measurement interval had often been set to every 4–6 h, some reports showed that there was no fixed standard and that the amount of GRV should be judged on a daily basis. Furthermore, the GRV measure may increase the risk of feeding tube occlusion, inappropriate discontinuation of EN, and the incidence of complications due to the decreased amount of EN administration [10, 20].

To address the aforementioned issues, SCCM/ASPEN guidelines 2016 suggested not to include the GRV monitoring as part of daily care [21]. In clinical practice, however, several institutions still use GRV as one of the criteria to confirm intestinal intolerance and to determine EN continuation or interruption. Metheny et al. [22] reported that more than 97% of nurses assessed intolerance solely by measuring GRVs. Notably, it is important to clarify the standards for each institution and to avoid inappropriate interruption and discontinuation of EN when GRV is within 500 mL. At our institution, we set a GRV of < 300 mL/day as a guide for EN intolerance; if GRV is over 300 mL/day, we administer rikkunshito to improve gastric emptying.

A longer duration of intestinal rest can prolong the attenuation of gastrointestinal peristalsis. Since inappropriate fasting or EN discontinuation may induce the prolongation and deterioration of paralytic ileus, it is important to minimize the fasting duration for diagnostic and treatment procedures. Patient intolerance had been reported to be one third of the reason for EN interruption [23], but true intolerance represents only half of this [21, 23]. Therefore, appropriate diagnosis of intestinal tolerance by the medical staff may reduce unnecessary discontinuation and withdrawal of EN.

As mentioned above, there is no useful and recommended method to monitor intestinal intolerance. During enteral nutrition management, it is important to always observe all gastrointestinal symptoms well and to confirm that these symptoms do not aggravate.

### Initiation of EN and EN protocol

EN is safe and suitable to administer for patients with mild or moderate paralytic ileus, as long as the patient’s hemodynamic status is stable [24]. The rate of achievement of the target amount of EN within 72 h was reported to be 30 to 85%, when EN was started after stabilization of hemodynamics, even before confirming the presence of peristaltic sounds. On the other hand, Kozar et al. reported that this rate reached 70 to 85% of the target amount of EN, when the EN protocol according to the circumstance of each facility was used [25]. The criteria to judge hemodynamic stabilization varies among facilities. At our institution, our criteria include small required administration dose of inotropic agents (e.g., ≤ 0.1 μg/kg/min of norepinephrine) or when the inotropic agents can be reduced.

### Increase of EN amount and intestinal tolerance

The use of an EN protocol should be recommended to achieve the target amount of EN [8]. The factors to be set in the protocol are diverse (Table 1), but it was reported that the rate of achievement of the EN target amount increased when the ICU staff used a protocol that accounted for these factors [10, 25–29]. After early EN initiation, the next step is to determine whether EN can be increased systematically to reach the target amount. In general, in critically ill patients, the amount of EN administered within the first week is set within about 80% of the target amount [30]. When actively increasing the amount of EN, creation of a protocol that is suitable for the actual situation at each facility is desirable and should take into account the other protocols reported in the past [31, 32]. However, the aim should be the adjustment of the EN amount along with the protocol, not to observe the protocol rigidly and inappropriately increasing the EN amount [33, 34]. Development of gastrointestinal disorders due to an inappropriate increase in EN amount or flow rate to achieve the target amount may lead to necessary interruption of EN administration and would take time to achieve the target amount by careful re-initiation of EN with a small amount. Therefore, slow start and continuous administration of EN, with delayed increase and decrease of EN amount, may avoid the inappropriate interruption and discontinuation of EN, thereby enabling early achievement of the target amount.

**Table 1:** Factors to be considered in the protocol

1. Criteria, conditions, and contraindications for enteral nutrition initiation	
2. Route of infusion (gastric vs. jejunal/ postpyloric)	
3. Method of infusion (intermittent vs. continuous)	
4. Target amount of EN formula	
5. Selection of the type of EN formula	
6. Flow rate at initiation and changing the flow rate	
7. Evaluation of gastrointestinal intolerance (gastric residual volume or abdominal X-ray)	
8. Measures against complications (changing the method of infusion or type of EN formula)	
9. How to manage the route (tube flushing, etc.)	

### Measures to mitigate reflux and aspiration

During EN administration, the risk of reflux and aspiration should be evaluated and prevented, especially in high-risk patients. Aspiration is one of the most notable complications of EN. The high-risk factors for aspiration are shown in Table 2 [17]. The measures to decrease the risk of reflux and aspiration are described below.

**Table 2:** High-risk factors for aspiration

Inability to protect the airway	
Presence of a nasoenteric access device	
Mechanical ventilation	
Age >70 years	
Reduced level of consciousness	
Poor oral care	
Inadequate nurse to patient ratio	
Supine positioning	
Neurologic deficits	
Gastroesophageal reflux	
Transport out of the ICU	
Use of bolus intermittent EN	

#### Elevation the head of the bed (30° to 45°)

In critically ill patients, elevating the head of the bed is a measure without economic burden to decrease the risk of aspiration, not only during EN management, but also during artificial ventilation, and so on [35–37]. Compared with supine to semi-recumbent positions, elevation of the head of the bed at 30° to 45° was shown to significantly reduce the incidence of pneumonia [35]. Notably, in reality, the angle often remains less than 30°, even if the head of the bed is elevated; therefore, it is important to check the angle on a regular basis. Furthermore, the position management was reported to be thorough when the physician clearly instructed the angle [38]. However, it is important to be aware that prolonged elevation of the head of the bed during continuous EN administration may increase the risk of developing sacral pressure ulcers.

#### Switch to continuous administration

Intermittent infusion of EN was shown to increase the risk of aspiration pneumonia [34]. MacLeod et al. reported that although the incidence of infection and the amount of EN were not different between continuous and intermittent infusion, the ICU mortality significantly decreased with continuous infusion (7.4% vs. 13.9%) [39]. Other RCTs showed that compared with intermittent infusion, continuous infusion had similar outcomes, including the mortality, incidence of infection, and duration of hospital stay [40–44] but significantly earlier achievement of the target amount of EN [42]. Continuous infusion seems to alleviate the intolerance to EN. Therefore, switching to continuous infusion may become one of the measures to decrease the risk of reflux and aspiration in patients at high risk or those with intolerance to gastric EN. The incidence of vomiting may increase with continuous gastric infusion of EN, because GRV cannot be measured. Therefore, short interruption of EN infusion on a regular basis and measurement of GRV as needed are desirable to establish.

#### Agents, such as prokinetic drugs or narcotic antagonists, to promote motility

Administration of prokinetic drugs, such as metoclopramide or erythromycin, had been shown to improve gastric emptying and intestinal intolerance [45]. Five RCTs revealed the effects of metoclopramide or erythromycin, in comparison with those of placebo [46–50]. Of these, a meta-analysis of three RCTs [48–50] reported that the administration of prokinetic drugs decreased GRV [21]. Another study showed that both metoclopramide and erythromycin decreased GRV in a similar efficacy [51]. Moreover, compared with metoclopramide alone, combination therapy with metoclopramide and erythromycin significantly decreased GRV [52]. However, none of the studies showed differences in mortality and incidence of pneumonia between the two drugs. Prokinetic agents may be effective in patients with high risk of aspiration or those with intolerance to gastric EN. On the other hand, both metoclopramide and erythromycin had been associated with QT prolongation, which predisposes to cardiac arrhythmias [53, 54]. Furthermore, it should be kept in mind that metoclopramide had been associated with adverse complications of extrapyramidal symptoms and tardive dyskinesia and that erythromycin may promote unnecessary use of antibiotics; moreover, administration of erythromycin to improve gastrointestinal motility is not covered by insurance in Japan. Therefore, these drugs should be discontinued quickly if they do not prove to be effective.

Narcotic analgesics may suppress gastrointestinal peristalsis. Administration of naloxone via a gastric tube to antagonize this adverse effect may improve the incidence of ventilator-associated pneumonia, decrease GRV, and increase the amount of EN infusion [55]. Therefore, narcotic antagonists may reduce the risk of reflux or aspiration.

These drugs have not been shown to improve outcomes, including mortality, duration of mechanical ventilation, and duration of ICU stay. In addition, various agents are used to improve gastrointestinal peristalsis in Japan. In particular, rikkunshito and mosapride citrate to promote gastric emptying, daikenchuto and prostaglandin F2α to promote intestinal peristalsis, and sodium picosulfate and magnesium oxide to promote bowel movement have been used on the basis of their pharmacological effects and experience [8]; however, evidence on the effectiveness of these drugs is not enough.

#### Switch to jejunal access (postpyloric route)

Switching the EN infusion route from gastric access to small intestinal (postpyloric) access had been shown to reduce the incidence of reflux [56, 57], aspiration, and pneumonia [10, 58, 59]. A meta-analysis that included 12 RCTs [3, 10, 58–67] reported that compared with gastric EN, small intestinal EN significantly reduced the incidence of pneumonia [21]. Another meta-analysis that included 7 RCTs [58–60, 65–68] showed small intestinal EN increase of the EN amount [21]. Therefore, switching the EN infusion route from gastric access to small intestinal access may become one of the measures to decrease the risk of reflux and aspiration in high-risk patients or those with intolerance to gastric EN. However, improvement of the EN amount and gastric emptying by jejunal (duodenal) EN, compared with gastric EN, were limited to patients with high severity [68] and had little effect [69–71]. Moreover, early jejunal EN had been reported to not reduce the incidence of pneumonia and to increase mild gastric bleeding [67]. On the other hand, gastric EN had been shown to reduce the duration of ICU stay [3, 63] and the incidence of infectious complications [58, 61], compared with jejunal EN.

Several methods, including X-ray fluoroscopy, auscultation, endoscopy, and ultrasound, had been reported as the methods that can be used to guide postpyloric feeding tube insertion. Although the optimal position (jejunum vs. duodenum) of the tip of the tube is controversial, insertion to the jejunum beyond the Treitz ligament had been reported to decrease the intragastric countercurrent [72]. At our institution, nasojejunal feeding tube insertion is performed at bedside using a transoral endoscope; however, regardless of the method used for postpyloric tube insertion, a gastric feeding tube is easier to insert and enables early initiation of EN. Because postpyloric tube insertion can delay EN initiation [64], early EN initiation by gastric tube should be prioritized.

Therefore, initiation of EN with jejunal feeding needs not be routine and should be switched from an initial gastric access depending on the severity of the patients’ condition. Jejunal feeding should be considered in cases with jejunostomy constructed by open laparotomy, those with reflux via the gastric tube, and those with vomiting due to delayed gastric emptying despite several measures to mitigate the intolerance to gastric EN. The ESPEN guidelines strongly recommended selecting gastric access for EN initiation and to shift to postpyloric access when patients develop intolerance despite administration of prokinetic drugs or in those with high risk of aspiration [73].

For severe ARDS patients, long-duration prone position is recommended to prevent ventilator-induced lung injury [74, 75]. PROSEVA study showed that prolonged (16 h) prone-positioning sessions significantly decreased mortality [76]. In such cases, EN administration in the long-duration prone position is needed. We confirmed that there was no difference in the amount of gastric reflux, regardless of gastric or jejunal access for continuous EN administration (unpublished data).

### Criteria, classification, and evaluation of diarrhea

Diarrhea and constipation are common gastrointestinal symptoms. In critically ill patients, the control of bowel movement is extremely important for intensive care and management. Bacterial translocation due to the attenuation of gastrointestinal peristalsis and the stagnation of intestinal contents may cause sepsis or organ disorder [77, 78]. Furthermore, diarrhea itself may induce several problems and complications, as described later. During early EN administration, observation of the status and control of bowel movement is important to achieve maximum effectiveness.

Although there are no definite criteria for diarrhea, indices, such as frequency of bowel movement > 3 to 5 times/day or a volume of bowel movement > 200–300 g/day, had been used commonly [79, 80]. In critically ill patients, various factors, including diseases; general condition; administration of drugs, such as antibiotics; presence or absence of EN administration; EN administration method; and the type of EN formula, may affect the property and volume of bowel movement. To definite the property and volume of bowel movement, evaluation tools, such as the Bristol stool form scale [81] or the King’s stool chart [82], have been used. Because critically ill patients have prolonged immobility and cannot assume a bowel movement posture similar to a healthy person, bowel movement is considered to be difficult unless the stool properties are softer than usual. Therefore, in critically ill patients, soft stools should be permitted while avoiding watery stools and bowel movement should be managed as Bristol scale 4 or higher.

**Fig. 1:**
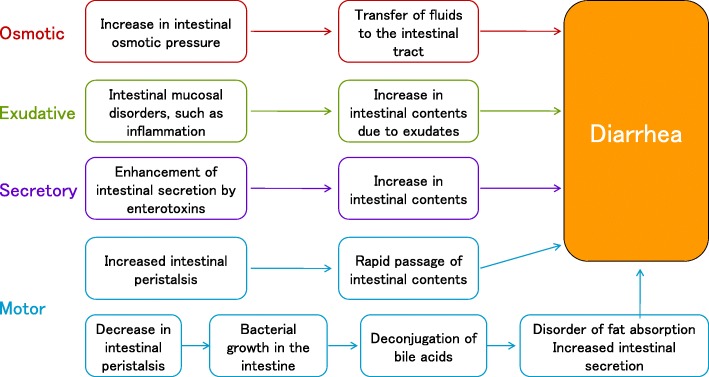
Classification and pathophysiology of diarrhea

Based on the pathologic features, diarrhea can be classified as osmotic, exudative, secretory, or motor [79, 80] (Fig. 1). In addition, diarrhea can also be classified as infectious or non-infectious, because treatment varies depending on the presence or absence of infection.

### Complications and problems associated with diarrhea

Prolonged diarrhea can cause deficiency in nutrient absorption, malnutrition, and increase in mortality [80]. As the incidence of complications increases, support with PN may be necessary. In addition, diarrhea may cause a decrease in the circulating blood volume; metabolic acidosis with loss of electrolytes and bicarbonate by excretion of large quantities of digestive juices; electrolyte abnormalities with loss of potassium, magnesium, and zinc; and contamination of surgical wounds and pressure ulcers [80, 83].

**Fig. 2:**
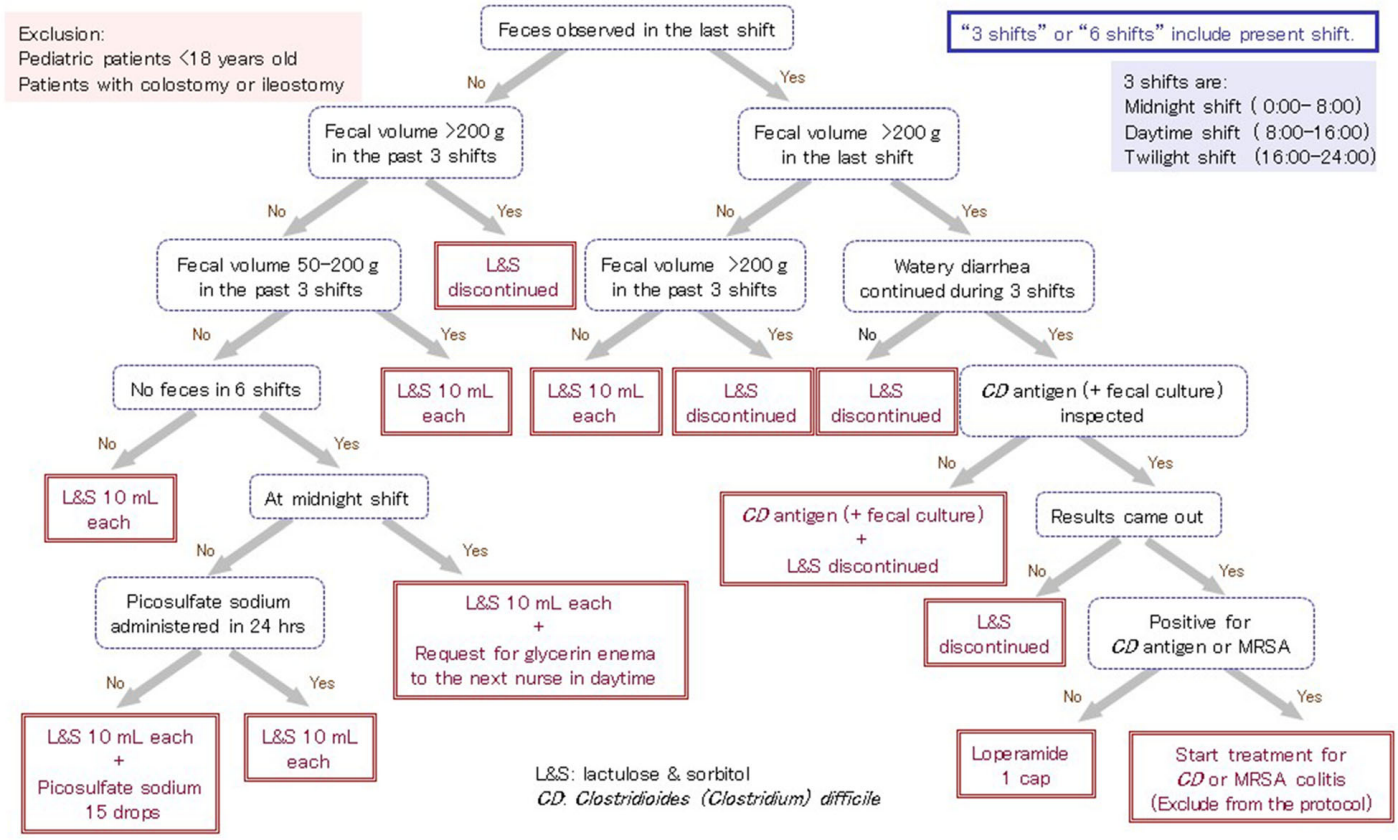
Protocol for fecal management (Sapporo Medical University Hospital ICU)

Strack van Schijndel et al. reported that a > 250-g/day volume of bowel movement may be used as an index of malnutrition [84]. Furthermore, Wierdsma et al. [85] reported that loss of nutrients in the feces increased as the volume of bowel movement increased and that daily measurement of bowel movement volume was important, because the risk of energy and protein deficiency may increase in patients with a bowel movement volume if > 350 g/day. Restriction of bowel movement volume to some extent is important for EN management in critically ill patients, because prolonged diarrhea is directly associated with energy deficit or negative energy balance [86], and the resulting malnutrition may impair immune function, increase the risk for infectious complications, and increase mortality. Therefore, a protocol for fecal management (Fig. 2) should be constructed according to the actual circumstances of each institution, similar to the protocols of EN initiation and decreasing the risk for aspiration. Administration of prokinetic drugs to improve the symptoms of constipation may be included in the protocol [87].

### Causes of diarrhea

Since EN can maintain the structure and function of the gastrointestinal mucosa better than PN, the development of diarrhea may be suppressed. However, diarrhea often occurs after EN initiation, depending on the methods of administration, amount, flow rate, and type of EN formula. If diarrhea occurs in critically ill patients on EN management, it is important to determine the cause (Table 3). In particular, in critically ill patients, diarrhea due to the administration of antibiotics should be taken avoided. Various risk factors for *Clostridioides (Clostridium) difficile*-associated diarrhea, which is the most frequent cause, have reported (Table 4) [88–93]. Furthermore, the incidence of diarrhea varies according to the type of antibiotics; the risk is high for quinolones and cephalosporins and low for macrolides [88, 94].

**Table 3:** Causes/risk factors for diarrhea, other than enteral nutrition

Causes	
1. Overdose of hyperosmotic drug (sorbitol, etc.)	
2. Use of broad-spectrum antibiotics	
3. Pseudomembranous enteritis due to *Clostridioides (Clostridium) difficile*	
4. Intestinal infections (MRSA enteritis, CMV enteritis, etc.)	
5. Inflammatory bowel diseases	
6. Intestinal graft-versus-host disease after hematopoietic stem cell transplantation	
7. Use of anticancer agents	
Risk factors	
1. Fever or hypothermia	
2. Presence of infections	
3. Malnutrition or hypoalbuminemia	
4. Sepsis or multiple organ failure	
5. Open-feed container	
6. Previous total parenteral nutrition

**Table 4:** Risk factors *Clostridioides (Clostridium) difficile*-associated diarrhea

Recent or current antibiotic therapy	
Prolonged stay in the ICU	
Use of PPIs	
Gender (more frequent in women)	
Severity of underlying diseases	
Enteral nutrition (especially in postpyloric feeding)	

### Diarrhea and EN

The incidence of EN-related diarrhea was reported to be the same, regardless of the administration route (gastric vs. postpyloric) [66, 95]. However, in theory, diarrhea had been thought to be more likely to occur with the direct injection of hyperosmotic EN formula into the jejunum than into the stomach. In many cases, diarrhea can be actually improved by converting jejunal EN to gastric EN. With regard to the method of EN administration, continuous infusion with the use of EN pump was reported to suppress the development of diarrhea, compared with intermittent infusion [41, 73, 96], but this effect was difficult to assess after the diarrhea has occurred [41, 96].

The factors related to the development of diarrhea are the content and composition of EN nutrients, such as carbohydrates, presence or absence and type of lipid, type of nitrogen source, presence or absence of lactose and milk protein, presence or absence of dietary fiber, and osmotic pressure. However, evidence on the effects of the contents and composition of EN nutrients had been insufficient. Most of the available studies were based on the investigation of the EN formula, not if the single ingredient.

### Prevention and treatment of diarrhea

The general symptomatic treatment of diarrhea comprises the administration of opioids and anticholinergic drugs and fluid replacement. If the cause of diarrhea does not seem to be EN and the presumed cause is being managed appropriately, EN should not be stopped unnecessarily and should be continued even in small amounts. The measures to prevent EN-related diarrhea are shown in Table 5.

**Table 5:** Measures to prevent EN-related diarrhea

At EN initiation	Start with a small amount and gradually increase
Flow rate of EN infusion	Switch intermittent infusion to continuous infusion
Route of EN feeding	Switch jejunal feeding to gastric feeding
Use of drugs	1. Detailed adjustment of agents that improve gastrointestinal peristalsis or laxative 2. Administration of herbal medicine or antidiarrheal drugs (after ruling out the other causes of diarrhea)
Changing the type of EN formula	1. Contains dietary fibers 2. Hypoosmotic 3. Does not contain fat, lactose, or milk protein 4. Contains peptide as nitrogen source (oligomeric diet)
Semisolidification of EN formula	1. Change to an EN formula (Hine E-Gel^®^) that can semisolidify in the stomach 2. Change to viscosity-adjusted liquid food (Meiflow^®^, etc.) 3. Add a thickener (REF-P1^®^) to make it semisolid in the gastrointestinal tract 4. Change to a semisolid type EN formula (patient with gastrostomy).

Elemental diet, in which the nitrogen source is formulated as amino acids, is hyperosmotic and can easily cause diarrhea. On the other hand, oligomeric diet, in which the nitrogen source is formulated as peptide, may not easily cause diarrhea, but the evidence on its efficacy had been insufficient. In Japan, the available nutrition products in medicine are Twinline^®^ NF (EN Otsuka Pharmaceutical Co., Ltd., Iwate, Japan) and Aminoleban^®^ EN (Otsuka Pharmaceutical Co., Ltd., Tokyo, Japan), and the available high-density liquid diet in food are Peptino^®^ (Terumo, Tokyo, Japan), Peptamen^®^ (Nestlé Health Science, Tokyo, Japan), and Hine E-Gel^®^ (Otsuka Pharmaceutical Factory, Inc., Naruto, Japan). In some cases, changing to an EN formula without lactose, milk protein, and lipid may improve the diarrhea. Especially in patients who have undergone surgery, such as pancreatoduodenectomy, changes in digestive enzyme secretion may alter the function of digestion and absorption and cause diarrhea.

Water-soluble dietary fibers, compared with insoluble dietary fibers, are more effective in preventing diarrhea. In particular, pectin and guar gum increase the viscosity, delay the excretion from the stomach and absorption in the small intestine, and decrease the flow of gastrointestinal contents by resisting against gastrointestinal peristalsis. Dietary fiber-enriched EN formula is usually administered to attenuate diarrhea and to improve constipation; among these, pectin had been reported to highly and effectively prevent diarrhea [97]. Since increasing the viscosity of gastrointestinal contents improves diarrhea, a semi-solid type of EN formula may be effective in patients with gastrostomy. In critically ill patients, however, the EN formula is difficult to make into a semi-solid form, because it is infused through a thin-diameter feeding tube. Recently, Hine E-Gel^®^ had become commercially available; it is a high-density liquid diet that includes pectin and changes into a gel form by its reaction with gastric acid and can undergo semisolidification in the stomach, even when administered through a thin-diameter tube. Moreover, viscosity-adjusted liquid food, such as Meiflow^®^ (Meiji Co., Ltd., Tokyo, Japan), which can be infused through a thin-diameter tube, can also prevent diarrhea. In contrast, one study reported that administration of dietary fiber-enriched EN formula had no efficacy in critically ill patients [98]. In addition, there is not enough evidence on the effectiveness of pre-/pro-/synbiotics in maintaining the bacterial flora. Further research is required.

In recent years, fecal microbiota transplantation had been carried out for several diseases, such as pseudomembranous enteritis and inflammatory bowel diseases, and has drawn attention because of its effects of decreasing the frequency of defection and improvement of stool properties. However, evidence on critically ill patients has not been established. Nevertheless, fecal microbiota transplantation for critically ill patients should attract more attention in the future, because normalization of gastrointestinal function and the bacterial flora can prevent BT and suppress the onset of sepsis or organ failure [99, 100].

## Conclusion

Gastrointestinal complications associated with EN, particularly vomiting and diarrhea, were described. One of the best ways to success for EN management is to continue as long as possible without interruption and discontinuation of EN easily by appropriate measures, even if gastrointestinal complications occur.

## Abbreviations

BT: Bacterial translocation
EN: Enteral nutrition
ESPEN: European Society for Clinical Nutrition and Metabolism
GRV: Gastric residual volume
ICU: Intensive care unit
PN: Parenteral nutrition
RCT: Randomized controlled trial
SCCM/ASPEN: Society of Critical Care Medicine/American Society for Parenteral and Enteral Nutrition

## References

[1] Mutlu GM, Mutlu EA, Factor P (2003). Prevention and treatment of gastrointestinal complications in patients on mechanical ventilation. Am J Respir Med.

[2] McClave SA, Martindale RG, Vanek VW (2009). Guidelines for the provision and assessment of nutrition support therapy in the adult critically ill patient: Society of Critical Care Medicine (SCCM) and American Society for Parenteral and Enteral Nutrition (A.S.P.E.N.). JPEN J Parenter Enteral Nutr.

[3] Minard G, Kudsk KA, Melton S (2000). Early versus delayed feeding with an immune-enhancing diet in patients with severe head injuries. J Parenter Enter Nutr.

[4] Dvorak MF, Noonan VK, Belanger L (2004). Early versus late enteral feeding in patients with acute cervical spinal cord injury: a pilot study. Spine..

[5] Kompan L, Vidmar G, Spindler-Vesel A (2004). Is early enteral nutrition a risk factor for gastric intolerance and pneumonia?. Clin Nutr.

[6] Malhotra A, Mathur AK, Gupta S (2004). Early enteral nutrition after surgical treatment of gut perforations: a prospective randomised study. J Postgrad Med.

[7] Peck MD, Kessler M, Cairns BA (2004). Early enteral nutrition does not decrease hypermetabolism associated with burn injury. J Trauma.

[8] The Committee on Japanese Guidelines for Nutrition Support Therapy in the Adult and Pediatric Critically Ill Patients, Japanese Society of Intensive Care Medicine. Japanese Guidel ines for Nutrition Support Therapy in the Adult and Pediatric Critically Ill Patients. J Jpn Soc Intensive Care Med 2016;23:185–281 (in Japanese).

[9] Marik PE (2014). Enteral nutrition in the critically ill: myths and misconceptions. Crit Care Med.

[10] Taylor SJ, Fettes SB, Jewkes C (1999). Prospective, randomized, controlled trial to determine the effect of early enhanced enteral nutrition on clinical outcome in mechanically ventilated patients suffering head injury. Crit Care Med.

[11] Pinilla JC, Samphire J, Arnold C (2001). Comparison of gastrointestinal tolerance to two enteral feeding protocols in critically ill patients: a prospective, randomized controlled trial. J Parenter Enter Nutr.

[12] Montejo JC, Miñambres E, Bordejé L (2010). Gastric residual volume during enteral nutrition in ICU patients: the REGANE study. Intensive Care Med.

[13] Tarling MM, Toner CC, Withington PS (1997). A model of gastric emptying using paracetamol absorption in intensive care patients. Intensive Care Med.

[14] Landzinski J, Kiser TH, Fish DN (2008). Gastric motility function in critically ill patients tolerant vs intolerant to gastric nutrition. J Parenter Enter Nutr.

[15] Cohen J, Aharon A, Singer P (2000). The paracetamol absorption test: a useful addition to the enteral nutrition algorithm?. Clin Nutr.

[16] McClave SA, Lukan JK, Stefater JA (2005). Poor validity of residual volumes as a marker for risk of aspiration in critically ill patients. Crit Care Med.

[17] McClave SA, DeMeo MT, DeLegge MH (2002). North American summit on aspiration in the critically ill patient: consensus statement. J Parenter Enter Nutr.

[18] Reignier J, Mercier E, Le Gouge A (2013). Effect of not monitoring residual gastric volume on risk of ventilator- associated pneumonia in adults receiving mechanical ventilation and early enteral feeding: a randomized controlled trial. JAMA..

[19] Poulard F, Dimet J, Martin-Lefevre L (2010). Impact of not measuring residual gastric volume in mechanically ventilated patients receiving early enteral feeding: a prospective before-after study. JPEN J Parenter Enteral Nutr.

[20] Powell KS, Marcuard SP, Farrior ES (1993). Aspirating gastric residuals causes occlusion of small-bore feeding tubes. JPEN J Parenter Enteral Nutr.

[21] McClave SA, Taylor BE, Martindale RG (2016). Guidelines for the Provision and Assessment of Nutrition Support Therapy in the Adult Critically Ill Patient: Society of Critical Care Medicine (SCCM) and American Society for Parenteral and Enteral Nutrition (A.S.P.E.N.). JPEN J Parenter Enteral Nutr.

[22] Metheny NA, Stewart BJ, Mills AC (2012). Blind insertion of feeding tubes in intensive care units: a national survey. Am J Crit Care.

[23] McClave SA, Sexton LK, Spain DA (1999). Enteral tube feeding in the intensive care unit: factors impeding adequate delivery. Crit Care Med.

[24] Martindale RG, Maerz LL (2006). Management of perioperative nutrition support. Curr Opin Crit Care.

[25] Kozar RA, McQuiggan MM, Moore EE (2002). Postinjury enteral tolerance is reliably achieved by a standardized protocol. J Surg Res.

[26] Barr J, Hecht M, Flavin KE (2004). Outcomes in critically ill patients before and after the implementation of an evidence-based nutritional management protocol. Chest..

[27] Martin CM, Doig GS, Heyland DK (2004). Southwestern Ontario Critical Care Research Network. Multicentre, cluster-randomized clinical trial of algorithms for critical-care enteral and parenteral therapy (ACCEPT). CMAJ..

[28] Adam S, Batson S (1997). A study of problems associated with the delivery of enteral feed in critically ill patients in five ICUs in the UK. Intensive Care Med.

[29] Spain DA, McClave SA, Sexton LK (1999). Infusion protocol improves delivery of enteral tube feeding in the critical care unit. JPEN J Parenter Enteral Nutr.

[30] Heyland DK, Stephens KE, Day AG (2011). The success of enteral nutrition and ICU-acquired infections: a multicenter observational study. Clin Nutr.

[31] Rice TW, Mogan S, Hays MA (2011). Randomized trial of initial trophic versus full-energy enteral nutrition in mechanically ventilated patients with acute respiratory failure. Crit Care Med.

[32] Rice TW, Wheeler AP, Thompson BT, National Heart, Lung, and Blood Institute Acute Respiratory Distress Syndrome (ARDS) Clinical Trials Network (2012). Initial trophic vs full enteral feeding in patients with acute lung injury: the EDEN randomized trial. JAMA..

[33] Mentec H, Dupont H, Bocchetti M (2001). Upper digestive intolerance during enteral nutrition in critically ill patients: frequency, risk factors, and complications. Crit Care Med.

[34] Ibrahim EH, Mehringer L, Prentice D (2002). Early versus late enteral feeding of mechanically ventilated patients: results of a clinical trial. J Parenter Enter Nutr.

[35] Drakulovic MB, Torres A, Bauer TT (1999). Supine body position as a risk factor for nosocomial pneumonia in mechanically ventilated patients: a randomized trial. Lancet..

[36] van Nieuwenhoven CA, Vandenbroucke-Grauls C, van Tiel FH (2006). Feasibility and effects of the semirecumbent position to prevent ventilator associated pneumonia: a randomized study. Crit Care Med.

[37] Metheny NA, Clouse RE, Chang YH (2006). Tracheobronchial aspiration of gastric contents in critically ill tube-fed patients: frequency, outcomes, and risk factors. Crit Care Med.

[38] Helman DL, Sherner JH, Fitzpatrick TM (2003). Effect of standardized orders and provider education on head-of-bed positioning in mechanically ventilated patients. Crit Care Med.

[39] MacLeod JB, Lefton J, Houghton D (2007). Prospective randomized control trial of intermittent versus continuous gastric feeds for critically ill trauma patients. J Trauma.

[40] Bonten MJ, Gaillard CA, van der Hulst R (1996). Intermittent enteral feeding: the influence on respiratory and digestive tract colonization in mechanically ventilated intensive-care-unit patients. Am J Respir Crit Care Med.

[41] Steevens EC, Lipscomb AF, Poole GV (2002). Comparison of continuous vs intermittent nasogastric enteral feeding in trauma patients: perceptions and practice. Nutr Clin Pract.

[42] Hiebert JM, Brown A, Anderson RG (1981). Comparison of continuous vs intermittent tube feedings in adult burn patients. J Parenter Enter Nutr.

[43] Kocan MJ, Hickisch SM (1986). A comparison of continuous and intermittent enteral nutrition in NICU patients. J Neurosci Nurs..

[44] Ciocon JO, Galindo-Ciocon DJ, Tiessen C (1992). Continuous compared with intermittent tube feeding in the elderly. JPEN J Parenter Enteral Nutr.

[45] Booth CM, Heyland DK, Paterson WG (2002). Gastrointestinal promotility drugs in the critical care setting: a systematic review of the evidence. Crit Care Med.

[46] Yavagal DR, Karnad DR, Oak JL (2000). Metoclopramide for preventing pneumonia in critically ill patients receiving enteral tube feeding: a randomized controlled trial. Crit Care Med.

[47] Berne JD, Norwood SH, McAuley CE (2002). Erythromycin reduces delayed gastric emptying in critically ill trauma patients: a randomized, controlled trial. J Trauma.

[48] Chapman MJ, Fraser RJ, Kluger MT (2000). Erythromycin improves gastric emptying in critically ill patients intolerant of nasogastric feeding. Crit Care Med.

[49] Reignier J, Bensaid S, Perrin-Gachadoat D (2002). Erythromycin and early enteral nutrition in mechanically ventilated patients. Crit Care Med.

[50] Nursal TZ, Erdogan B, Noyan T (2007). The effect of metoclopramide on gastric emptying in traumatic brain injury. J Clin Neurosci.

[51] MacLaren R, Kiser TH, Fish DN (2008). Erythromycin vs metoclopramide for facilitating gastric emptying and tolerance to intragastric nutrition in critically ill patients. JPEN J Parenter Enteral Nutr.

[52] Nguyen NQ, Chapman M, Fraser RJ (2007). Prokinetic therapy for feed intolerance in critical illness: one drug or two?. Crit Care Med.

[53] Al-Khatib SM, LaPointe NM, Kramer JM (2003). What clinicians should know about the QT interval. JAMA..

[54] Li EC, Esterly JS, Pohl S (2010). Drug-induced QT-interval prolongation: considerations for clinicians. Pharmacotherapy..

[55] Meissner W, Dohrn B, Reinhart K (2003). Enteral naloxone reduces gastric tube reflux and frequency of pneumonia in critical care patients during opioid analgesia. Crit Care Med.

[56] Lien HC, Chang CS, Chen GH (2000). Can percutaneous endoscopic jejunostomy prevent gastroesophageal reflux in patients with preexisting esophagitis?. Am J Gastroenterol.

[57] Heyland DK, Drover JW, MacDonald S (2001). Effect of postpyloric feeding on gastroesophageal regurgitation and pulmonary microaspiration: results of a randomized controlled trial. Crit Care Med.

[58] Hsu CW, Sun SF, Lin SL (2009). Duodenal versus gastric feeding in medical intensive care unit patients: a prospective, randomized, clinical study. Crit Care Med.

[59] Acosta-Escribano J, Fernández-Vivas M, Grau Carmona T (2010). Gastric versus transpyloric feeding in severe traumatic brain injury: a prospective, randomized trial. Intensive Care Med.

[60] Montecalvo MA, Steger KA, Farber HW (1992). Nutritional outcome and pneumonia in critical care patients randomized to gastric versus jejunal tube feedings. The Critical Care Research Team. Crit Care Med.

[61] Kortbeek JB, Haigh PI, Doig C (1999). Duodenal versus gastric feeding in ventilated blunt trauma patients: a randomized controlled trial. J Trauma.

[62] Day Lisa, Stotts Nancy A., Frankfurt Anna, Stralovich-Romani Annette, Volz Monica, Muwaswes Marylou, Fukuoka Yoshimi, OʼLeary-Kelley Colleen (2001). Gastric Versus Duodenal Feeding in Patients With Neurological Disease: A Pilot Study. Journal of Neuroscience Nursing.

[63] Davies AR, Froomes PR, French CJ (2002). Randomized comparison of nasojejunal and nasogastric feeding in critically ill patients. Crit Care Med.

[64] White H, Sosnowski K, Tran K (2009). A randomised controlled comparison of early post-pyloric versus early gastric feeding to meet nutritional targets in ventilated intensive care patients. Crit Care.

[65] Kearns PJ, Chin D, Mueller L (2000). The incidence of ventilator-associated pneumonia and success in nutrient delivery with gastric versus small intestinal feeding: a randomized clinical trial. Crit Care Med.

[66] Montejo JC, Grau T, Acosta J (2002). Multicenter, prospective, randomized, single-blind study comparing the efficacy and gastrointestinal complications of early jejunal feeding with early gastric feeding in critically ill patients. Crit Care Med.

[67] Davies AR, Morrison SS, Bailey MJ (2012). ENTERIC Study Investigators; ANZICS Clinical Trials Group. A multicenter, randomized controlled trial comparing early nasojejunal with nasogastric nutrition in critical illness. Crit Care Med.

[68] Huang HH, Chang SJ, Hsu CW (2012). Severity of illness influences the efficacy of enteral feeding route on clinical outcomes in patients with critical illness. J Acad Nutr Diet.

[69] Ho KM, Dobb GJ, Webb SA (2006). A comparison of early gastric and post-pyloric feeding in critically ill patients: a meta-analysis. Intensive Care Med.

[70] Marik PE, Zaloga GP (2003). Gastric versus post-pyloric feeding: a systematic review. Crit Care.

[71] Heyland DK, Drover JW, Dhaliwal R (2002). Optimizing the benefits and minimizing the risks of enteral nutrition in the critically ill: role of small bowel feeding. J Parenter Enter Nutr.

[72] Minard G (1994). Enteral access. Nutr Clin Pract.

[73] Singer Pierre, Blaser Annika Reintam, Berger Mette M., Alhazzani Waleed, Calder Philip C., Casaer Michael P., Hiesmayr Michael, Mayer Konstantin, Montejo Juan Carlos, Pichard Claude, Preiser Jean-Charles, van Zanten Arthur R.H., Oczkowski Simon, Szczeklik Wojciech, Bischoff Stephan C. (2019). ESPEN guideline on clinical nutrition in the intensive care unit. Clinical Nutrition.

[74] Mentzelopoulos SD, Roussos C, Zakynthinos SG (2005). Prone position reduces lung stress and strain in severe acute respiratory distress syndrome. Eur Respir J.

[75] Galiatsou E, Kostanti E, Svarna E (2006). Prone position augments recruitment and prevents alveolar overinflation in acute lung injury. Am J Respir Crit Care Med.

[76] Guérin C, Reignier J, Richard JC (2013). Prone positioning in severe acute respiratory distress syndrome. N Engl J Med.

[77] Alexander JW, Boyce ST, Babcock GF (1990). The process of microbial translocation. Ann Surg.

[78] Mainous MR, Ertel W, Chaudry IH (1995). The gut: a cytokine-generating organ in systemic inflammation?. Shock.

[79] Whelan K, Judd PA, Preedy VR (2004). Enteral feeding: the effect on faecal output, the faecal microflora and SCFA concentrations. Proc Nutr Soc.

[80] Wiesen P, Van Gossum A, Preiser JC (2006). Diarrhoea in the critically ill. Curr Opin Crit Care.

[81] O'Donnell LJ, Virjee J, Heaton KW (1990). Detection of pseudodiarrhoea by simple clinical assessment of intestinal transit rate. BMJ..

[82] Whelan K, Judd PA, Taylor MA (2004). Assessment of fecal output in patients receiving enteral tube feeding: validation of a novel chart. Eur J Clin Nutr.

[83] Kelly TW, Patrick MR, Hillman KM (1983). Study of diarrhea in critically ill patients. Crit Care Med.

[84] Strack van Schijndel RJ, Wierdsma NJ, van Heijningen EM (2006). Fecal energy losses in enterally fed intensive care patients: an explorative study using bomb calorimetry. Clin Nutr.

[85] Wierdsma NJ, Peters JH, Weijs PJ (2011). Malabsorption and nutritional balance in the ICU: fecal weight as a biomarker: a prospective observational pilot study. Crit Care.

[86] Villet S, Chiolero RL, Bollmann MD (2005). Negative impact of hypocaloric feeding and energy balance on clinical outcome in ICU patients. Clin Nutr.

[87] Oczkowski SJW, Duan EH, Groen A (2017). The use of bowel protocols in critically ill adult patients: a systematic review and meta-analysis. Crit Care Med.

[88] Modena S, Bearelly D, Swartz K (2005). Clostridium difficile among hospitalized patients receiving antibiotics: a case–control study. Infect Control Hosp Epidemiol.

[89] Dial S, Alrasadi K, Manoukian C (2004). Risk of Clostridium difficile diarrhea among hospital inpatients prescribed proton pump inhibitors: cohort and case–control studies. Can Med Assoc.

[90] Crabtree TD, Pelletier SJ, Gleason TG (1999). Clinical characteristics and antibiotic utilization in surgical patients with Clostridium difficile-associated diarrhea. Am Surg.

[91] Vesta KA, Wells PG, Gentry CA (2005). Specific risk factors for Clostridium difficile-associated diarrhea: a prospective, multicenter, case control evaluation. Am J Infect Control.

[92] Kyne L, Sougioultzis S, McFarland LV (2002). Underlying disease severity as a major risk factor for nosocomial Clostridium difficile diarrhea. Infect Control Hosp Epidemiol.

[93] Bliss DZ, Johnson S, Savik K (1998). Acquisition of Clostridium difficile and Clostridium difficile-associated diarrhea in hospitalized patients receiving tube feeding. Ann Intern Med.

[94] Yip C, Loeb M, Salama S (2001). Quinolone use as a risk factor for nosocomial Clostridium difficile-associated diarrhea. Infect Control Hosp Epidemiol.

[95] Meert KL, Daphtary KM, Metheny NA (2004). Gastric vs small-bowel feeding in critically ill children receiving mechanical ventilation: a randomized controlled trial. Chest..

[96] Lee JS, Auyeung TW (2003). A comparison of two feeding methods in the alleviation of diarrhoea in older tube-fed patients: a randomised controlled trial. Age Ageing.

[97] Schultz AA, Ashby-Hughes B, Taylor R (2000). Effects of pectin on diarrhea in critically ill tube-fed patients receiving antibiotics. Am J Crit Care.

[98] Yang G, Wu XT, Zhou Y (2005). Application of dietary fiber in clinical enteral nutrition: a meta-analysis of randomized controlled trials. World J Gastroenterol.

[99] Klingensmith NJ, Coopersmith CM (2016). Fecal microbiota transplantation for multiple organ dysfunction syndrome. Crit Care.

[100] McClave SA, Patel J, Bhutiani N (2018). Should fecal microbial transplantation be used in the ICU?. Curr Opin Crit Care.

